# Multicomponent shell traits are consistent with an individual recognition function of the appearance of common murre (*Uria aalge*) eggs: A biological replication study

**DOI:** 10.1002/ece3.7264

**Published:** 2021-02-14

**Authors:** Rebecca L. Ducay, Alec B. Luro, Erpur S. Hansen, Mark E. Hauber

**Affiliations:** ^1^ Department of Evolution, Ecology, and Behavior School of Integrative Biology University of Illinois Urbana‐Champaign Urbana IL USA; ^2^ South Iceland Nature Research Centre Vestmannaeyjar Iceland

**Keywords:** alcid, perceptual modeling, recognition systems, vision

## Abstract

In dense breeding colonies, and despite having no nest structure, common murres (or guillemots: *Uria aalge*) are still able to identify their own eggs. Each female murre's egg is thought to be recognized individually by the shell's avian‐perceivable traits. This is because the eggshells’ visible traits conform to expectations of the identity‐signaling hypothesis in that they show both high intraindividual repeatability and high interindividual variability. Identity signaling also predicts a lack of correlation between each of the putative multicomponent recognition traits, yielding no significant relationships between those eggshell traits that are generated by mutually exclusive physiological factors. Using a multivariate analysis across eggshell size and shape, avian‐perceivable background coloration, spot (maculation) shape, and spot density, we detected no unexpected statistical correlations between Icelandic common murre egg traits lacking known physiological or mathematical relationships with one another. These results biologically replicate the conclusions of a recent eggshell trait study of Canadian common murres using similar methodology. We also demonstrate the use of static correlations to infer identity signaling function without direct behavioral observations, which in turn may also be applied to rare or extinct species and provide valuable insight into otherwise unknown communicative and behavioral functions.

## Graphical abstract

A multivariate analysis of eggshells taken from an Icelandic population of common murre (or guillemot: *Uria aalge*) reveals that no unexpected correlations exist between avian‐perceivable eggshell traits. These results biologically replicate the conclusions of a recent eggshell trait study of Canadian common murres using the same methodology, as well as provide further evidence for the functionality of avian‐perceivable eggshell traits as identity signals in the common murre.

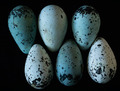

## 1 INTRODUCTION

Investment in the care of dependent and vulnerable offspring can require great expenditure of parental resources (Clutton‐Brock, 1991). Thus, the ability of the parent to recognize its own (genetic) young, rather than unrelated one(s), is adaptive because it ensures that care has a positive influence on parental fitness (Sherman et al., 1997).

**Figure 1:**
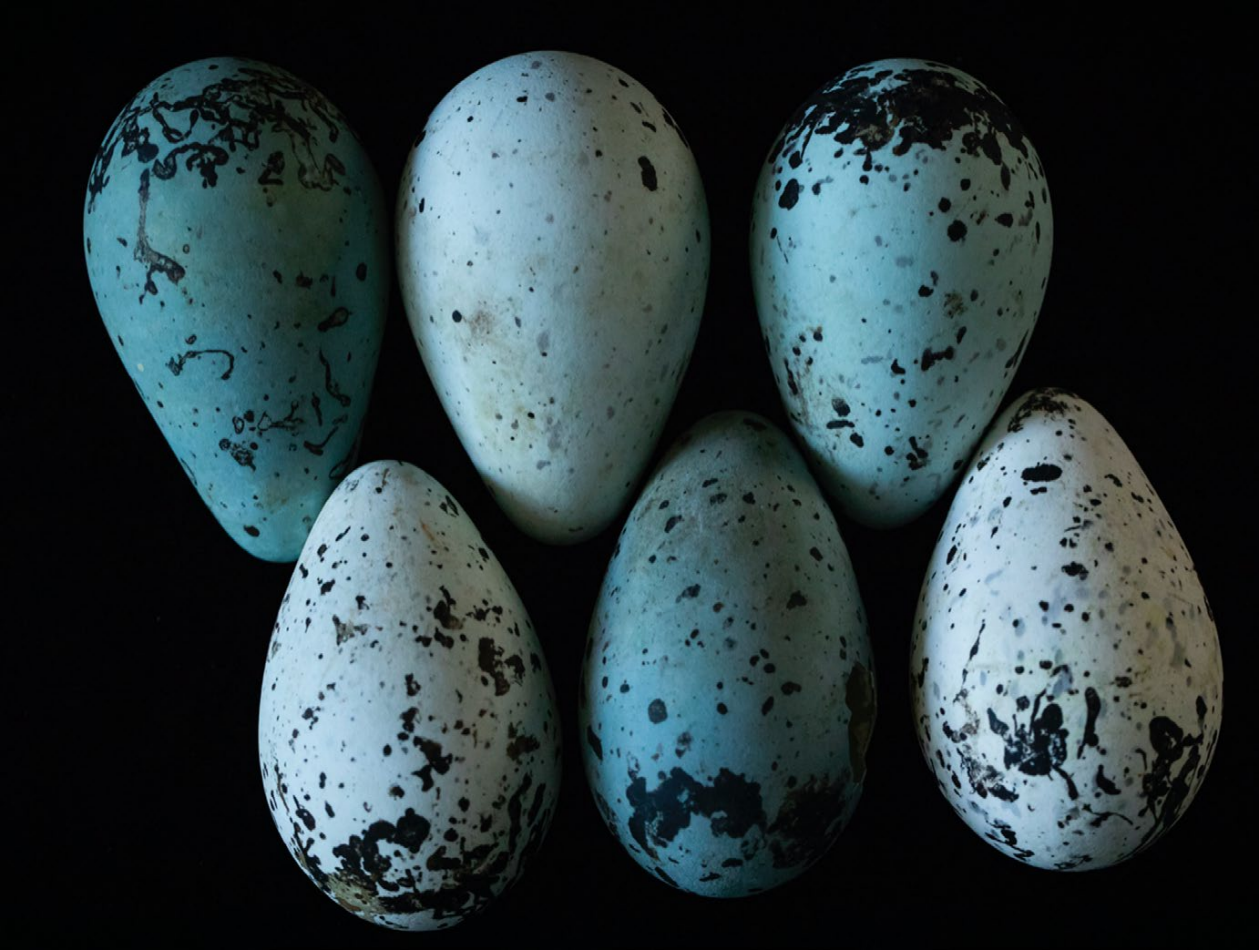
Representative eggshells of the common murre's (*Uria aalge*) from Iceland, collected in 2014. Photo credit: B. Stauffer

Natural selection for direct offspring recognition is thought to be especially influential in species that breed in large, densely packed colonies where indirect positional information is diminished (Tibbetts & Dale, 2007). Under such circumstances, individual recognition may subserve offspring identification as it allows an individual (the parent) to recognize another (its offspring) based on the latter's distinctive trait(s) (Dale et al., 2001). One textbook‐example avian species (e.g., Faaborg, 2020) that breeds in large, dense colonies and does not build a nest, is the common murre (or guillemot: *Uria aalge*), which recognizes its own single egg such that parents can distinguish it from other, nearby eggs (Tschanz, 1959, Tschanz, 1968). In turn, common murres are also known for their human‐ and avian‐perceivably distinct individual eggshell traits (Figure 1), including high interindividual variation in maculation density, color, and shape, background coloration, and shell size and shape (Birkhead Thompson & Biggins, 2017; Birkhead, Thompson, Jackson, et al., 2017; Dale, 2006; Hauber, Bond, et al., 2019). Specifically, each single egg laid by the same female common murres across repeated breeding attempts (whether from the same or different seasons) is typically consistent in many of these same eggshell traits, including shell coloration, spotting pattern, egg size (Hauber, Luro, et al., 2019), and egg shape (Birkhead, Thompson, & Biggins, 2017).

High interindividual variation in perceivable traits between focal subjects is a diagnostic property of identity signals; that is, traits used to communicate about and recognize a specific individual (Tibbetts & Dale, 2007). Because of this, it is suspected that many of the variable common murre eggshell traits may be identity signals and, thus, play a critical role in the accurate egg recognition abilities of murre parents (Hauber, Bond, et al., 2019; Quach et al., 2020). In comparison, the common murre's sister species, the thick‐billed murre (*Uria lomvia*), that breeds in less densely packed colonies has lower avian‐perceivable egg trait variabilities between individuals (Quach et al., 2020) and, in turn, poorer behavioral egg recognition than that of the common murre (Gaston et al., 1993).

Another notable quality of identity signals that may distinguish them from other types of recognition cues is the predicted lack of correlation among the multiple modalities or components of the same individual's identity signals, which is due to the negatively frequency‐dependent selection by which these types of multi‐modal or multicomponent signals evolve (Tibbetts & Dale, 2007). Accordingly, Hauber, Bond, et al. (2019) recently determined that different avian‐perceivable traits of the common murre eggshells’ background coloration and a physical metric of its maculation density were not statistically related within the same egg specimens. Thus, these traits may be suitable candidates for identity signals in this species’ eggs.

Here, we used a geographically independent set of egg samples from an Icelandic breeding population (Hauber, Luro, et al., 2019) to partially replicate (sensu the “partial replication” definition of Kelly, 2006) the findings of Hauber, Bond, et al. (2019) on Canadian common murres. Specifically, we expanded on published findings by assessing additional correlations between various avian‐perceivable eggshell traits that had been found to have high intraindividual repeatability in our prior study (i.e., shell background coloration, spot density, spot shape, and egg size: Hauber, Luro, et al., 2019) and in another published study (egg shape: Birkhead, Thompson, & Biggins, 2017).

Methodological replication has long been regarded as an important necessity in all fields of science, for it helps to curb the possibility of random error in various stages of experimentation and statistical analyses (Kelly, 2006; Rosenthal, 1990). Indeed, the field of behavioral ecology has seen many instances in which statistically significant relationships were reported in one publication that could not be supported by replicative studies (e.g., as discussed in Seguin & Forstmeier, 2012). Here, we wished to replicate Hauber, Bond, et al. (2019) not out of concerns of possible errors; rather, we considered that our methodologies had been sound and, thus, we applied them to other sources of eggshell samples and morphological traits with high interindividual variability and intraindividual repeatability. We aimed to reveal confirmatory and, perhaps, even additional evidence for the classification of common murre eggshell appearance as a source of individual identity signals. In accordance with the expectations under identity signaling (Tibbetts & Dale, 2007) in general, and the results of Hauber, Bond, et al. (2019) in particular, we predicted that a multivariate analysis would reveal no statistical correlations between any eggshell traits with no clear underlying physiological or mathematical relationships (a priori summarized in Table 1).

**Table 1:** Predicted physiological and mathematical correlations between common murre eggshell characteristics

Predicted correlation	Association (direction)
Spot size (mm^2^) ∝ Spot aspect (w/l)	Mathematical (negative). Both traits are derived from the same raw measurements of spot width and length.
Spot Size (mm^2^) ∝ Spot Density (1/cm)	Mathematical (positive). The larger the average spot size, the greater the likelihood for a given spot to intersect the long axis. Physiological (positive). Protoporphyrin, the pigment specifically responsible for avian eggshell's brown hues and maculation patterns (Verdes et al., 2015), can be deposited in both larger spots and/or greater numbers of spots (Brulez et al., 2014).
Egg volume (mm^3^) ∝ Egg elongation (w/l)	Mathematical (positive). Both traits are derived from the same raw measurements of shell width and length.

## 2 METHODS

### 2.1 Sample collection

Common murre's eggs (*N* = 84) used in our new analyses were collected during a single week from different incubating individuals for a commercial purpose (public sale allowed under Icelandic laws) on the south Langanes Cliffs of Northeastern Iceland (66°18'41.2''N, 14°49'15.9''W), and obtained by us in the 2014 breeding season for scientific purposes. Eggs were all deemed to be first‐laid eggs of the season, and all had been incubated for no more than 50% of the incubation period by the murres prior to collection. The eggs were opened, their contents emptied, and washed with water in Iceland, before long‐term storage in a dark and room temperature environment. We imported these eggshells to the USA under both US Department of Agriculture (#125504) and US Fish and Wildlife (#MB50883B‐0) permits.

### 2.2 Egg measurements

Eggshell dimensions were taken as length and width, recorded by a caliper, to the nearest 0.1 mm. Egg length was defined as the longest distance between the sharp and blunt poles of the egg, and egg width was defined as the widest distance perpendicular to the longitudinal axis. These measurements were then used to calculate egg volume (as a proxy: width^2^ * length) and egg elongation (width/length).

For each egg, we also collected length and width measurements from the three human‐assessed largest maculations (spots, lines, and/or squiggles) that were again recorded using a caliper (to the nearest 0.1 mm). We acknowledge that using calipers to measure linear dimensions on a curved surface may have introduced some unaccounted confounds into our measurements. The length of each spot was defined as its longest dimension and width was measured as the longest dimension perpendicular to that longitudinal axis. For line/squiggle‐type maculation, we used the longest linear distance for length and the second longest linear distance perpendicular to the longest axis for width. These measurements were averaged for each egg to generate an average maculation length and width. Post hoc comparisons confirm that, critically, both maculation width and length across the three raw metrics were repeatable within each eggshell (both *p* < .0001, *R^2^* > 0.5). These values were then used to find the average spot size for each egg (width * length) and the average spot aspect (width/length).

Spot density was determined for each egg by counting the number of distinct (i.e., human‐visibly disconnected) maculations along the long axis of the egg and dividing the resulting number by the length of that egg. We did not use digital data or other software‐based approaches to analyze eggshell maculation patterns in our study because none of the published spot‐based metrics are based, sensu stricto, on measurement‐driven avian visual‐physiology derived perceptual modeling (unlike coloration analyses, see below), despite their increasing use for such purposes (e.g., Stoddard et al., 2014, Gómez and Liñán‐Cembrano, 2016, Šulc et al., 2020).

### 2.3 Background coloration and avian perceptual modeling

Avian‐perceivable spectral reflectance (300–700 nm) of common murre eggshell backgrounds were recorded using an Ocean Optics USB2000 spectrometer under the illumination of a DT mini‐lamp (Ocean Optics, Inc.). Reflectance measurements were taken from unobscured portions of background coloration from each egg to exclude areas with maculation. Spectral reflectance measurements were relative to a 99% Spectralon reflectance standard (Labsphere Inc., North Sutton NH) and taken with a probe at a 90° angle (following Igic et al., 2012) in triplicate for each egg.

**Figure 2:**
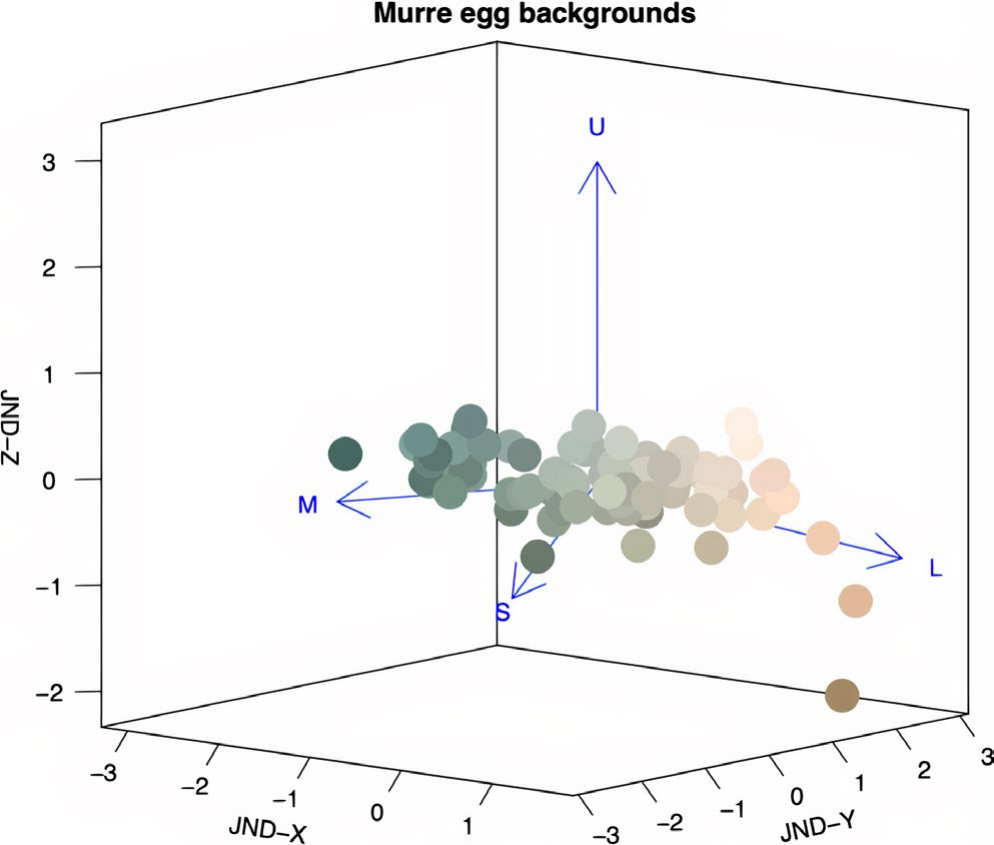
Chromatic just noticeable difference (JND) XYZ coordinate plot. Each point represents a single common murre's (*Uria aalge*) eggshell background coloration. All points are mean‐centered, and directions indicated by the blue arrows correspond to the relative stimulation of the violet‐sensitive (U), shortwave‐sensitive (S), medium‐wave‐sensitive (M), and long‐wave‐sensitive (L) photoreceptors by the eggs’ coloration. Different hues show the human‐visible colors of each of the analyzed murre eggshells’ background generated from respective reflectance spectra

We used a receptor‐noise‐limited visual model to quantify avian perceived color discriminability (Vorobyev and Osorio, 1998) between the background spectral reflectance of the collected eggs. Visual modeling was done using the *vismodel* and *coldist* functions of the *pavo* package version 2.4.0 (Maia et al., 2019) in R 4.0.0 (R Core Team, 2017). Though the actual light wavelength sensitivities of the common murre's photoreceptors are unknown, the species likely possesses a violet‐sensitive (VS) rather than ultraviolet‐sensitive (UVS) vision based on the molecular sequence of the SWS1 opsin gene (Ödeen et al., 2013). Thus, approximate common murre visual perception was modeled using the VS visual modeling approach described in Hauber, Bond, et al. (2019), under the same assumptions that the eggs were illuminated in full sunlight (*D65*) and that perceived color differences were independent of viewing background appearance (*ideal* background). Avian‐perceivable differences in chromaticity, as chromatic just noticeable differences (JNDs), were generated from the cone photoreceptors’ quantum catch values (violet‐sensitive (U), shortwave‐sensitive (S), medium‐wave‐sensitive (M), and long‐wave‐sensitive (L)) for murre egg colors and mapped into 3‐dimensional coordinates using the *jndxyz* function of the *pavo* package. The central origin point in 3D space was set as the mean murre egg color, and Euclidean distances from the origin for each egg were set to be equal to the JNDs from the mean egg color (Figure 2). This method produced X, Y, and Z coordinates for egg background coloration in 3D space of distances measured in units of JND (see Hauber, Bond, et al., 2019).

### 2.4 Statistical analyses

The data used for our analyses have been deposited for open access as specified in the Data Accessibility section below. To test for correlations between visible eggshell traits, we used the multivariate and correlations function in JMP 14.2.0. (SAS Institute), with each egg representing a single unit of biological analysis (*N* = 84). The variables analyzed were as follows: eggshell elongation (w/l), eggshell volume (mm^3^), average spot size (mm^2^), average spot aspect (spot w/l), spot density (1/cm), and the X, Y, and Z coordinate of JND values of avian‐perceivable shell background coloration. Specifically, a restricted maximum‐likelihood (REML) estimation method was used due to the relatively small sample size of the dataset and the presence of three missing values in spot aspect and density (due to immaculate eggs). Though the three JND coordinates collectively represent a "bluegreen‐white‐brown" axis of avian‐perceivable coloration (Hanley et al., 2015), the biological implications of each of these individual JND values remain unknown and, thus, correlations between the X, Y, and Z coordinates were also excluded from our analysis. No values were used to assign weight or frequency to any of the listed variables. This method produced Pearson product–moment correlations between all the analyzed variables. The Bonferroni method was used to adjust (lower) the probability of type 1 statistical errors due to multiple comparisons (adjusted α = 0.002) (Bland & Altman, 1995, but see Nakagawa, 2004).

## 3 RESULTS

**Figure 3:**
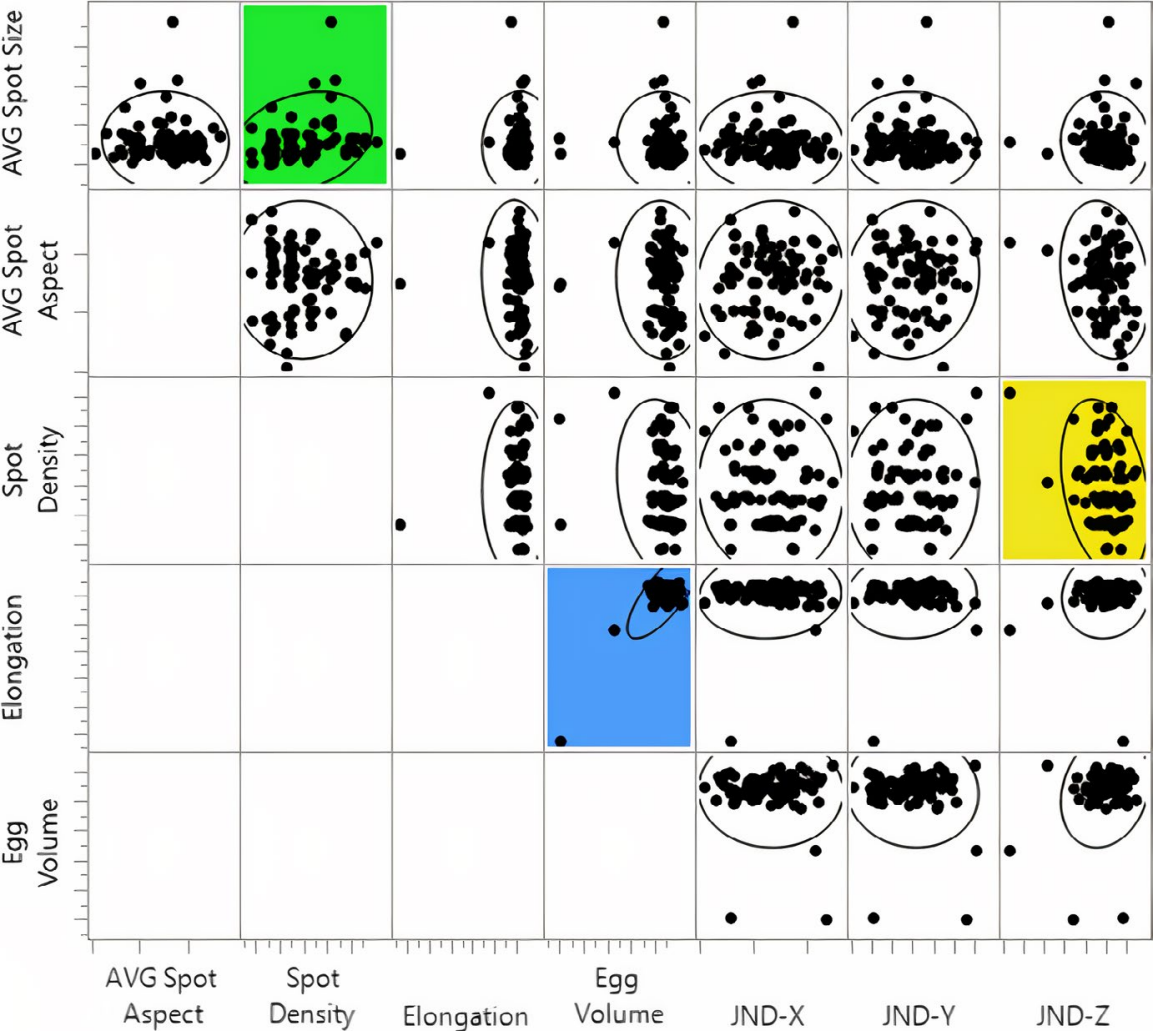
Multivariate scatterplot matrix with density ellipses. Predicted significant correlation (*p* < .002) is marked in blue, predicted nonsignificant correlation (0.002 < *p* < .05) is marked in green, and unpredicted nonsignificant correlation (.002 < *p* < .05) is marked in yellow

The multivariate analysis generated *n* = 25 separate correlations, and upon inspection, we found no significant relations between most (96%) of the variables examined (|*R*| < 0.2, *p* > .002), except for one (4%) significant correlation in which egg volume was positively correlated to egg elongation (*R* = 0.825, *p* < .0001) (Table 2). Even though average spot size was also found to be positively related to spot density (*R* = 0.273, *p* = .0119) and spot density was negatively related to the Z coordinate of shell background coloration JND (*R* = −0.301, *p* = .0054), neither of these correlations were statistically significant according to our adjusted α level (Table 2). A visual representation of all the tested relationships is included in Figure 3.

**Table 2:** Pearson product–moment correlation R‐values given by multivariate analysis

	Spot aspect (w/l)	Spot density (1/cm)	Egg elongation (w/l)	Egg volume (mm^3^)	JND‐X	JND‐Y	JND‐Z
Spot size (mm^2^)	−0.028	**0.273**	0.053	−0.014	−0.043	−0.04	−0.028
Spot aspect (w/l)		−0.011	−0.059	−0.05	0.078	0.095	−0.197
Spot density (1/cm)			−0.084	−0.173	−0.049	0.028	**−0.301**
Egg elongation (w/l)				**0.825**	−0.052	−0.067	0.155
Egg volume (mm^3^)					−0.022	−0.058	0.195

Note

Correlations of *p* < .05 are bolded while correlations of *p* < .002 are bolded and underlined.

## 4 DISCUSSION

The single statistically significant correlation between egg volume and egg elongation produced in our analysis occurred between trait values a priori predicted to be related (Table 1). Thus, as expected under the identity signaling hypothesis (Tibbetts & Dale, 2007), we found no statistically significant correlations between murre eggshell traits with mutually exclusive determinative factors. Specifically, our results confirm the lack of detected correlation between background coloration and spot density, as reported by Hauber, Bond, et al. (2019) using common murre eggshells sampled from geographically independent (Icelandic versus Canadian) populations. Further, in support of individual recognition cue signaling theory, as applied to common murre eggs (e.g., Dale, 2006), our findings also provide evidence for the lack of correlation between other separate eggshell traits that had previously been demonstrated to have high interannual repeatability for known females, including spot size, spot shape, and egg size (Hauber, Luro, et al., 2019), as well as egg shape (Birkhead, Thompson, & Biggins, 2017).

Though not statistically significant after Bonferroni corrections (α = 0.002), there were two correlations in our dataset whose probability fell below the uncorrected α level of 0.05. One of these relationships, spot size by spot density (*R^2^* = 0.075), can be explained both mathematically and physiologically (Table 1). In contrast, the final relationship that we mentioned between spot density and the JND‐Z coordinate of avian‐perceivable background coloration (*R^2^* = 0.091) was unexpected, given that background JND coordinates have shown no correlation to maculation density in previous work (Hauber, Bond, et al., 2019). However, upon inspection (Figure 3) we discovered that this relationship has resulted because of a single egg in our sample, which was an outlier in both spot density and JND coordinate Z. In addition, this egg also represented an outlier in egg elongation and volume. The exclusion of this egg from the multivariate analysis diminishes the correlation between spot density and the JND‐Z coordinate (*R* = −0.171, *p* = .1218) while the other correlations throughout our dataset (Figure 3) continue to persist at the same levels of statistical significance. Inspection of this eggshell revealed no observable biological abnormalities, nor did human error appear to have misrepresented the unique features of this egg. We have, therefore, decided to keep this egg in the multivariate analysis of correlations since the intent of this study was to assess the correlation between murre eggshell characteristics, and this egg presents a possible, though rare, combination of eggshell characteristics. Nevertheless, even with the inclusion of this egg, we conclude that the resulting correlation was still the result of a false‐positive (type 1) statistical error, which is a probable occurrence when multiple (*n* = 25) comparisons are conducted, and no correction is applied to the standard α value; thus, we consider that the use of Bonferroni correction was appropriate to reduce the probability of type 1 errors in our dataset (but see Nakagawa, 2004; also below) and we report no significant correlations beyond what had been predicted in Table 1.

Bonferroni corrections have been criticized in the field of behavioral ecology as it is a conservative method that possesses a perhaps too‐high probability of producing false‐negative (type 2) statistical errors than other correction methods for multiple comparisons (Nakagawa, 2004). However, even with the uncorrected α value of 0.05, we did not detect more than two additional correlations. This is surprising, as there might be more correlations between eggshell background coloration traits and maculation traits within murre eggs. For example, previous studies suggest correlations between background coloration and maculation eggshell traits for the thick‐billed murre (Gaston & Nettleship, 1981; Quach et al., 2020). Additionally, there have been reports of a positive correlation between protoporphyrin and biliverdin concentrations in eggshells of different avian species (the two pigments responsible for aspects of eggshell coloration; Cassey et al., 2012). However, this positive correlation between the two pigments has not yet been examined extensively across eggs within the same species.

Our results reveal a lack of significant correlations between eggshell traits with no obvious physiological or mathematical relationships, as is theoretically expected for the outcome of negatively frequency‐dependent selection for identity signals (Tibbetts & Dale, 2007). Although we cannot confirm the interannual repeatability of eggshells traits within our Icelandic population given the lack of repeated sampling of the same individual females, we exclusively analyzed eggshell traits shown to be highly repeatable within individuals across breeding seasons in past studies (Birkhead, Thompson, & Biggins, 2017; Hauber, Luro, et al., 2019). In addition, all of the analyzed eggshell traits had also demonstrated high levels of interindividual variability in the published literature (Birkhead, Thompson, & Biggins, 2017; Birkhead, Thompson, Jackson, et al., 2017; Dale, 2006; Hauber, Bond, et al., 2019). Given that identity signals are expected to be both highly variable between individuals and possess a high degree of genetic determination within individuals, along with a lack of correlation between their multicomponent signals (Tibbetts & Dale, 2007), we consider our new results here to provide further evidence for the functionality of maculation shape, spot density, eggshell size and shape, and avian‐perceivable background coloration as possible identity signals for common murre eggs (Quach et al., 2020).

Further evaluations of eggshell trait correlations for avian (and other) species with differing selection pressures and recognition capabilities are necessary in order to assess the robustness of this methodology for predicting possible identity signals. For instance, the related razorbill (*Alca torda*) is a colony‐nesting seabird which displays poor egg recognition abilities (Birkhead, 1978) and, thus, razorbill eggshells would not be expected to possess the characteristics of negatively frequency‐dependent selection recognition trait distribution (Tibbetts & Dale, 2007). Without these selection pressures, then we may expect a higher degree of association between visible eggshell characteristics of species without egg recognition abilities, in comparison with species that do possess these abilities (Quach et al., 2020).

Behavioral recognition experiments using experimental eggs are necessary to determine whether the murre eggshell traits truly function as signals of identity across diverse and distinct populations. Tschanz (1968) provided experimental evidence for individual egg recognition in one population of this species, though his experimental methods were not designed to discern which characteristics of the eggshells allowed for the recognition by the incubating parent. Future experimentation involving parental retrieval versus rejection of common murre and related species’ eggs (e.g., Gaston et al., 1993) with specifically manipulated natural or model eggshell traits (e.g., Igic et al., 2015) within the same incubation site could provide the needed causal connection between individual recognition and eggshell trait variation in these colonial seabirds.

Finally, direct behavioral observations are not always possible when analyzing the recognition capabilities of species, such as species which are rare, elusive, or extinct. The ability to infer the existence of identity signals from biological trace evidence, such as remaining eggshells, could provide valuable insight into possible behavioral characteristics of species that cannot be directly observed. One species to which eggshell analyses similar to those performed here could be applied is the great auk (*Pinguinus impennis*) (e.g., Birkhead et al., 2020), which diverged from the common murre about 17 mya (Smith & Clarke, 2015). Few behavioral observations were recorded prior to the extinction of the great auk, and thus, no evidence exists for or against the great auk's nesting and, perhaps, individual egg recognition abilities (Bengtson, 1984). Analysis of the few remaining preserved eggshells of the great auk, including the quantification of variability and correlation between observable eggshell characteristics, could thus serve to assist in the reconstruction of the otherwise unknown recognition behaviors of this (and other) extinct species with variably colored and maculated eggshells (Birkhead et al., 2020; Fuller, 1999).

## 5 CONCLUSIONS

The designation of common murre eggshell traits as identity signals involves the confirmation that these traits fit the predicted characteristics of negatively frequency‐dependent selection on multicomponent recognition traits (Tibbetts & Dale, 2007). Past studies provided evidence that common murres’ eggshell maculation density and shape, background coloration, and eggshell size and shape have high interindividual variability (Birkhead, Thompson, & Biggins, 2017; Birkhead, Thompson, Jackson, et al., 2017; Dale, 2006; Hauber, Bond, et al., 2019) and high intraindividual repeatability for known females, suggesting genetic determination (Birkhead, Thompson, & Biggins, 2017; Hauber, Luro, et al., 2019). The results of our multivariate analysis of the listed eggshell traits complement support these findings as no unexpected significant correlations were detected. Though functional identity signaling should preferably be verified by direct experimental evidence, the designation of identity signals through the analysis of remnant materials, such as eggshells of extinct species, may also provide critical insight into the life histories of rare, elusive, or extinct species that cannot be observed or experimented upon directly (Birkhead et al., 2020).

## CONFLICT OF INTERESTS

We declare no competing interests.

## AUTHOR CONTRIBUTIONS

**Rebecca Lynn Ducay:** Data curation (equal); Formal analysis (equal); Writing‐original draft (lead); Writing‐review & editing (equal). **Alec Luro:** Formal analysis (equal); Software (equal); Visualization (equal); Writing‐review & editing (equal). **Erpur Snær Hansen:** Data curation (equal); Formal analysis (equal); Writing‐review & editing (equal). **Mark Hauber:** Conceptualization (lead); Data curation (equal); Formal analysis (equal); Funding acquisition (equal); Methodology (equal); Project administration (lead); Writing‐original draft (supporting); Writing‐review & editing (equal).

## Data Availability

The data are available at Figshare.com through the https://doi.org/10.6084/m9.figshare.13625762
